# Serum Apurinic/Apyrimidinic Endodeoxyribonuclease 1 (APEX1) Level as a Potential Biomarker of Cholangiocarcinoma

**DOI:** 10.3390/biom9090413

**Published:** 2019-08-26

**Authors:** Doungdean Tummanatsakun, Tanakorn Proungvitaya, Sittiruk Roytrakul, Temduang Limpaiboon, Sopit Wongkham, Chaisiri Wongkham, Atit Silsirivanit, Ongart Somintara, Sakkarn Sangkhamanon, Siriporn Proungvitaya

**Affiliations:** 1 Centre of Research and Development of Medical Diagnostic Laboratories (CMDL), Faculty of Associated Medical Sciences, Khon Kaen University, Khon Kaen 40002, Thailand; 2 National Center for Genetic Engineering and Biotechnology (BIOTEC), National Science and Technology Development Agency (NSTDA), Pathumthani 12120, Thailand; 3 Cholangiocarcinoma Research Institute (CARI), Faculty of Medicine, Khon Kaen University, Khon Kaen 40002, Thailand; 4 Department of Biochemistry, Faculty of Medicine, Khon Kaen University, Khon Kaen 40002, Thailand; 5 Department of Surgery, Faculty of Medicine, Khon Kaen University, Khon Kaen 40002, Thailand; 6 Department of Pathology, Faculty of Medicine, Khon Kaen University, Khon Kaen 40002, Thailand

**Keywords:** cholangiocarcinoma, secretome, bioinformatics, apurinic/apyrimidinic endodeoxyribonuclease 1 (APEX1), metastasis

## Abstract

Diagnostic and/or prognostic biomarkers for cholangiocarcinoma (CCA) are still insufficient with poor prognosis of patients. To discover a new CCA biomarker, we constructed our secretome database of three CCA cell lines and one control cholangiocyte cell line using GeLC-MS/MS. We selected candidate proteins by five bioinformatics tools for secretome analysis. The inclusion criteria were as follows: having predicted signal peptide or being predicted as non-classically secreted protein; together with having no transmembrane helix and being previously detected in plasma and having the highest number of signal peptide cleavage sites. Eventually, apurinic/apyrimidinic endodeoxyribonuclease 1 (APEX1) was selected for further analysis. To validate APEX1 as a bio-marker for CCA, serum APEX1 levels of 80, 39, and 40 samples collected from CCA, benign biliary diseases (BBD), and healthy control groups, respectively, were measured using dot blot analysis. The results showed that serum APEX1 level in CCA group was significantly higher than that in BBD or healthy control group. Among CCA patients, serum APEX1 level was significantly higher in patients having metastasis than in those without metastasis. The higher level of serum APEX1 was correlated with the shorter survival time of the patients. Serum APEX1 level might be a diagnostic and prognostic biomarker for CCA.

## 1. Introduction

Cholangiocarcinoma (CCA) is cancer originated from biliary epithelial cells. The incidence of CCA is very high in northeastern Thailand [1], especially where people live close to the water reservoir and consume cyprinoid fish infected with metacercariae of the liver fluke, *Opisthorchis viverrini* (OV) [2]. In addition to the epidemiological co-incidence of liver fluke infection and CCA, animal model experiments of CCA genesis demonstrated that OV, together with nitrosamine carcinogen treatment, caused CCA [3,4]. Currently, several serum markers, such as carbohydrate antigen 19-9 (CA19-9), carcinoembryonic antigen (CEA), and alkaline phosphatase (ALP), have been used for diagnosis of CCA. However, the sensitivity and specificity of these markers are not quite satisfactory to detect CCA [3,5,6]. Therefore, it is necessary to discover novel markers to improve the efficacy of CCA diagnosis and prognosis.

Cancer secretome is a total protein released from cancer cells and/or tissues into extracellular microenvironment and is known as the reservoir of biomarkers, and secretome proteomics has extensively been used for discovery of cancer markers [7]. The proteins secreted from cancer cells are of particular interest because they mostly enter into the blood circulation and can be measured with minimally invasive assays [8]. In our previous study [9], using bioinformatics search for classically secreted proteins from the secretome data of four CCA cell lines, including KKU-OCA17, KKU-213, KKU-214, and KKU-100, we identified intercellular adhesion molecule 1 (ICAM-1) as a potential prognostic marker for CCA. Thus, as the first step of this study, we aimed to search not only classically secreted proteins but also non-classically secreted proteins using the secretome data of three CCA cell lines, KKU-213, KKU-214, and KKU-100, but not KKU-OCA17, to identify a diagnostic marker. This is because KKU-OCA17 was characterized as non-human CCA cell line after our previous study [9]. Eventually, we identified apurinic/apyrimidinic endodeoxyribonuclease 1 (APEX1) as the potential candidate of the diagnostic marker for further analysis.

APEX1 gene encodes a protein of 318 amino acids, with a molecular weight of 36.5 kDa [10]. The C-terminal domain is involved in base excision DNA repair under oxidative stress, and the N-terminal domain is important in protein reduction-oxidation function [11,12,13,14]. It is mainly localized in the nucleus, but nuclear and cytoplasmic co-localization has been reported in several cancers [15,16,17]. Moreover, Kim et al. recently reported that APEX1 could be a potential diagnostic marker for clear cell renal carcinoma and hepatobiliary carcinomas [17].

Therefore, the feasibility of APEX1 as a biomarker for CCA was validated further by measuring its level in the sera from CCA, benign biliary diseases (BBD), and healthy controls. Moreover, the functional importance of APEX1 for tumor metastasis was verified using in vitro cell migration and invasion assays of gene-silenced CCA cells. The results presented here showed that APEX1 could be not only a diagnostic marker for CCA but also be a prognostic marker for CCA.

## 2. Results

### 2.1. The Candidate Proteins from the Secretome Data

The secretomes of three CCA cell lines and their control immortalized cholangiocyte cell line named MMNK1 were quantitatively compared based on MS signal intensities using a DeCyderTM MS Differential Analysis Software (version 2.0, GE Healthcare, Piscataway, NJ, USA). Subsequently, the protein data and individual intensities were visualized with Mev software (version 4.6.1, Dana-Farber Cancer Institute, Boston, MA, USA).

In the MMNK1 secretome, 1000 total proteins were identified, whereas 996, 1010, and 1005 proteins were identified in the secretomes of KKU-100, KKU-213, and KKU-214, respectively. While 1117 proteins were shared with at least two cell lines, 11 proteins were found to be unique in MMNK1 secretome, one protein was unique in KKU-100 secretome, four proteins unique in KKU-213 secretome, and five proteins unique in KKU- 214 secretome.

**Figure 1:**
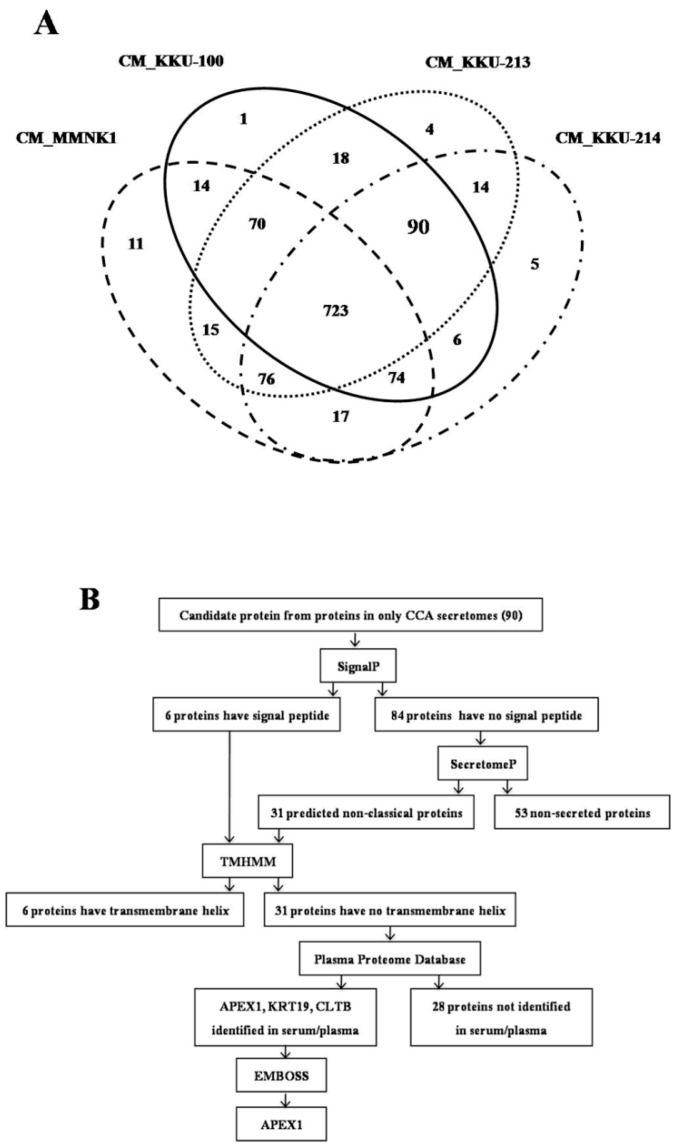
Data analysis from mass spectrometry and selection of candidate proteins. (**A**) A total of 1138 protein molecules were found in cholangiocarcinomas (CCAs) and immortalized cholangiocyte secretomes. Venn diagram presents the number of proteins in each cell line secretome sample and the degree of protein overlapping. The oval dashed line indicates the total proteins in the conditioned medium (CM) of MMNK1, oval solid line is the total proteins in CM_KKU-100, oval dotted line is total proteins in CM_KKU-213, and the broken line is the total proteins in CM_KKU-214. Ninety proteins were expressed commonly in three CCA secretomes. (**B**) Flowchart of the selection of secretory proteins from 90 proteins overexpressed in CCA. Proteins having a signal peptide or non-classical proteins that have no transmembrane helix were identified in serum/plasma database, and the maximum number of signal peptide cleavage sites was used for stepwise selection.

After Venn diagram analysis, there were 90 up-regulated proteins common in three CCA cell lines compared with MMNK1 by statistical significance at *p* < 0.05. All 90 up-regulated proteins have log2 fold increase >1 fold in MS signal intensities compared with their counterpart in MMNK1. To explore the CCA biomarkers, those 90 up-regulated proteins were subjected to a further selection of candidate proteins (Figure 1A). Besides, we provided MS data of 90 proteins, including the accession ID and the data of expression in three CCA cell lines (Table S1).

### 2.2. Bioinformatic Analyses to Select Potential Biomarkers for CCA 

Since 90 proteins were up-regulated commonly in the secretomes of three CCA cell lines compared with MMNK1 secretome, proteins having secretory protein nature were selected from those 90 CCA unique proteins according to the flowchart (Figure 1B). As the first step, using SignalP 4.0, 90 CCA unique proteins were divided into secretory and non-secretory proteins. Only six proteins having a signal peptide and cleavage sites were identified as the classical secretory proteins. Then, the remaining 84 proteins were analyzed using SecretomeP 2.0. The results showed that 31 of 84 proteins contained a signal peptide sequence, indicating that they are also likely to be the secretory proteins. Subsequently, for those 37 (6+31) potentially secretory proteins, the presence of a transmembrane helix was predicted using Transmembrane hidden Markov model (TMHMM) 2.0. Out of 37 proteins, six of them were identified to have a transmembrane helix. As the final step, for those 31 proteins lacking transmembrane helix, proteins present in serum or plasma were identified using the Plasma Proteome Database (PPD). Only three (APEX1, Keratin 19 (KRT19), and Clathrin light chain B (CLTB)) out of 31 proteins were identified to be present in serum or plasma. For these three proteins, the presence of signal peptide cleavage sites between a signal sequence and the mature exported protein was predicted using the European Molecular Biology Open Software Suite (EMBOSS). Then, the proteins that had the highest number of signal peptide cleavage sites were selected. As such, APEX1 was selected as a potential biomarker candidate for CCA. Fold increase of the MS signal intensities of APEX1 in the secretome of three cell lines, KKU-100, KKU-213, and KKU-214, compared with their control MMNK1 was 15.4, 15.3, and 15.1, respectively.

### 2.3. APEX1 Levels in the Serum Samples

**Figure 2:**
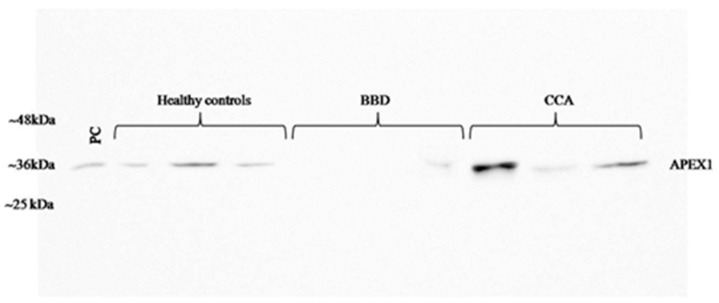
Validation of the selected candidate protein, APEX1 in serum samples by western blot analysis. BBD: benign biliary diseases.

**Figure 3:**
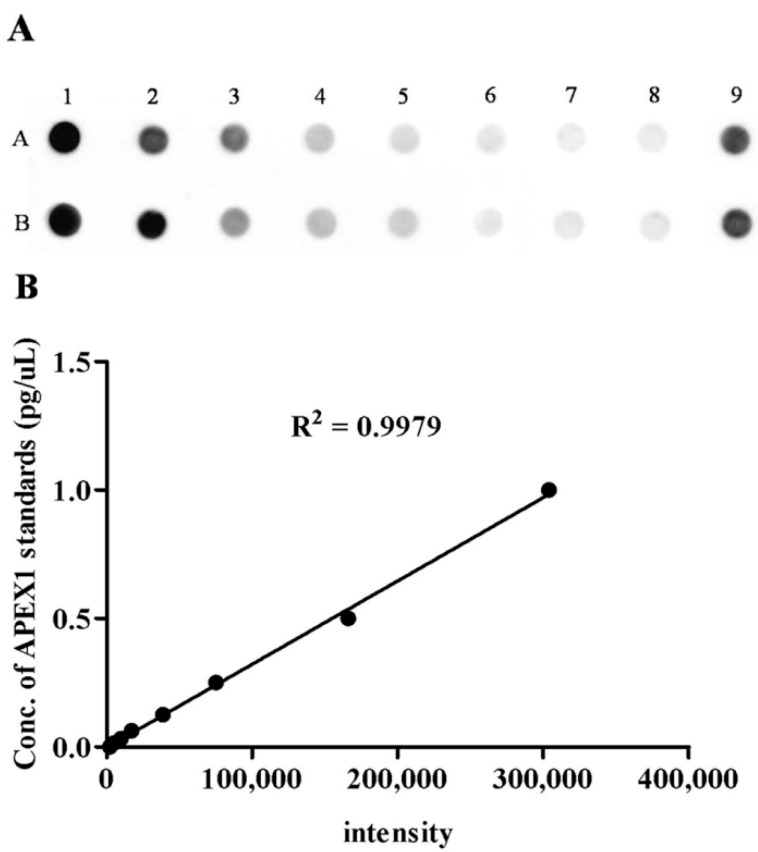
The level of serum APEX1 detected by dot blot assay. (**A**) The image of the dot blot assay of serum APEX1. Samples A1 to A7 were standards, sample A8 was the blank control, sample A9 was positive control, and row B was the duplicated row of A. (**B**) The standard curve of APEX1 levels.

**Figure 4:**
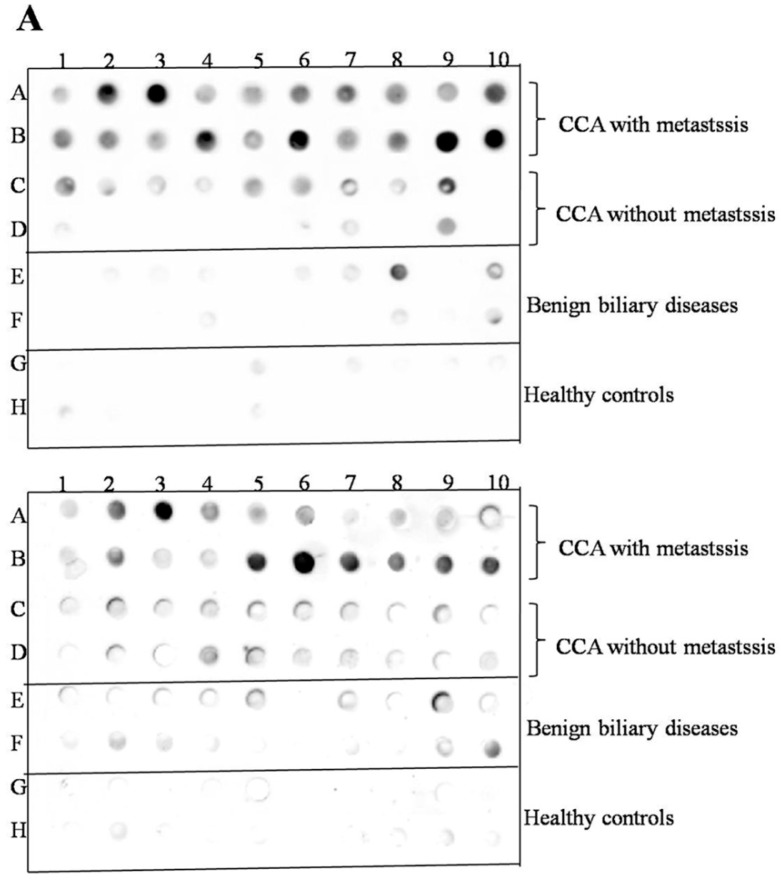

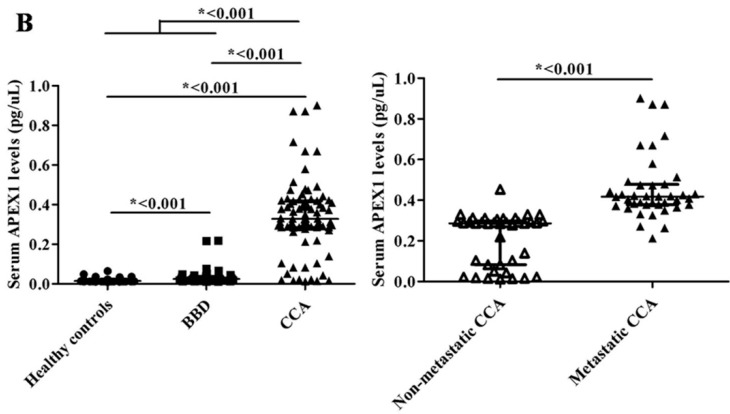
The validation of selected candidate protein, APEX1. (**A**) The result of dot blot. (**B**) The validation of serum APEX1 level as the biomarker using a dot blot assay. Long horizontal line: median value; short upper and lower lines: interquartile range. *: Statistically significant difference.

To validate the candidate protein APEX1 selected by proteomic analysis as a CCA marker, the expression of APEX1 protein in the representative sera from CCA, BBD, and control groups was examined using western blot analysis. The results revealed the clear presence of 36 kDa size band corresponding to positive control protein (Hela cell lysate) in the sera from all three groups. Moreover, APEX1 level in the sera of CCA patients appeared to be higher than that of BBD and healthy controls (Figure 2). Then, APEX1 levels of 80 serum samples from CCA patients, 39 from BBD patients, and 40 from healthy controls were measured semi-quantitatively using a dot blot assay system based on the standard curve created by using a standard APEX1 protein (Figure 3A,B). APEX1 level in the sera of CCA patients was significantly higher than that of BBD or healthy controls (Table 1, Figure 4A). 

To ensure the equivalence of protein dotting, serum samples were shuffled and randomly spotted onto the membrane (Figure S1A). When the results were compared with those of the results of the original experiment shown in the results, a linear correlation was observed between the first set and the second set of shuffled spotting (Figure S1B). To validate the accuracy of dot blot quantification, the correlation of the intensity between western blot and dot blot was examined using three selected serum samples (high, medium, and low expression of APEX1 in dot blot) of CCA patients. A linear correlation was observed between western blot and dot blot (Figure S2).

Then, we divided the CCA patients group into those having lymph node metastasis and those having no metastasis. Serum APEX1 level was higher in CCA patients having metastasis than in those having no metastasis (Figure 4B), suggesting that this protein might be used as a potential serum biomarker for discriminating metastatic and non-metastatic CCA patients.

To evaluate whether the serum APEX1 level can be used for the diagnostic biomarker for CCA, a receiver operating characteristic (ROC) curve was constructed by plotting sensitivity versus 100 –specificity or false positive rate. In ROC analysis between healthy control and BBD group, area under the ROC curve (AUC) was 0.748 at the cut-off value of 0.018, indicating the rather good diagnostic efficiency. In between CCA and BBD groups, AUC was 0.927 at a cut-off value of 0.050, which indicated good diagnostic efficiency. In between healthy control and CCA groups, AUC was 0.966, indicating good diagnostic efficiency at the cut-off value of 0.040. Moreover, in case of discrimination of CCA from pooled healthy persons and BBD, AUC was 0.947, which indicated the good diagnostic efficiency, at the cut-off value of 0.080 (Table 2).

Besides, the sensitivity and specificity values of APEX1 for CCA diagnosis were compared to those of currently used biomarkers, carbohydrate antigen 19-9 (CA19-9), carcinoembryonic antigen (CEA), and alkaline phosphatase (ALP). Diagnostic value of individual marker revealed that APEX1 had higher sensitivity and specificity than other markers. Furthermore, the combination of APEX1 and CA19-9, CEA, or ALP showed a remarkable improvement in specificity compared with each marker alone. Especially, the combination of all four markers, including APEX1, CA19-9, CEA, and ALP, showed prominent improvement of CCA detection with the high specificity of 99% (Table 3).

### 2.4. Correlation Between Serum APEX1 Level and the Clinicopathological Features of CCA Patients

**Figure 5:**
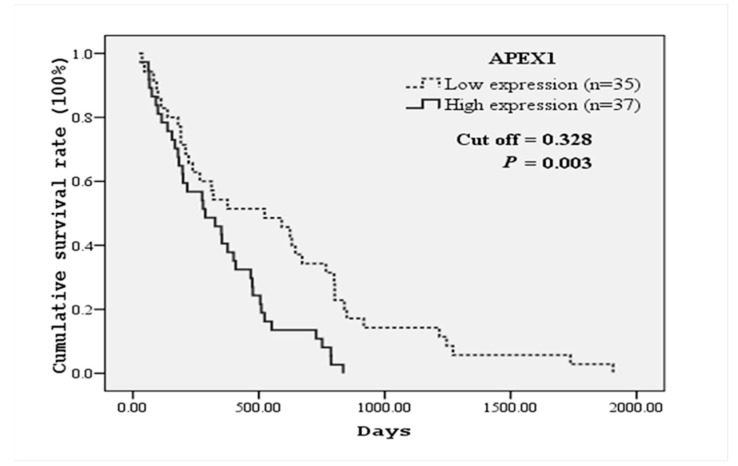
Kaplan–Meier survival curves of CCA patients based on serum APEX1 levels. CCA patients were divided into high and low serum APEX1 level groups using a median serum APEX1 value (0.328) of CCA patients as a cut-off. The curves show overall survival of CCA patients having high (solid line) and low (dashed line) serum APEX1 levels. A significant difference in the survival time was observed between high and low APEX1 level groups (log-rank test *p*-value = 0.003).

Because serum APEX1 level was significantly higher in CCA patients with lymph node metastasis than in the patients without metastasis, we further analyzed the correlation between serum APEX1 level and the clinicopathological features of CCA patients. For this purpose, we divided CCA patients into high and low serum APEX1 groups using a median APEX1 value of CCA patients as a cut-off. High serum APEX1 level was correlated with lymph node metastasis and shorter mean survival time, but not with other parameters (Table 4). Correlation between serum APEX1 level and the survival time was further confirmed using the Kaplan–Meier graph (Figure 5). High serum APEX1 group had a mean survival time of 337.2 days, whereas low serum APEX1 group had a mean survival time of 569.8 days with *p* = 0.003 by log-rank test.

### 2.5. Effect of APEX1 Gene Silencing on Cell Motilities of CCA Cell Line 

**Figure 6:**
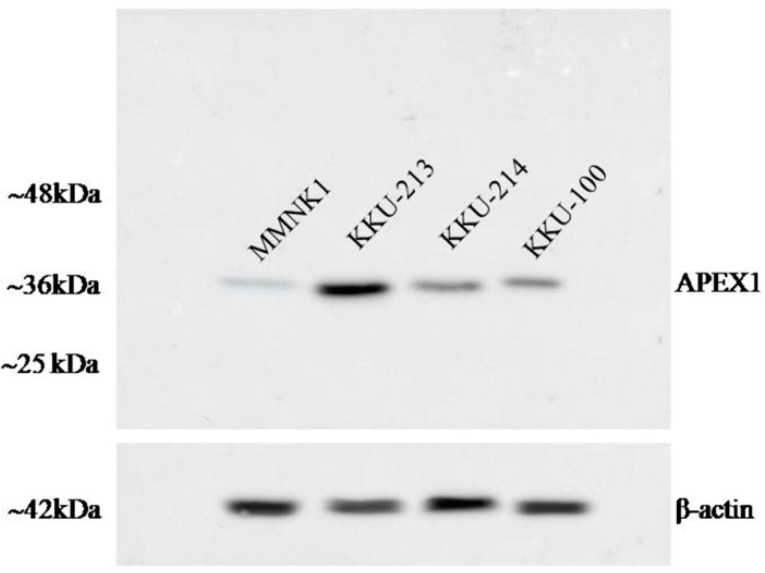
The image of western blot. The expression of APEX1 in three CCA cell lines and control immortalized cholangiocyte MMNK1 cell line.

**Figure 7:**
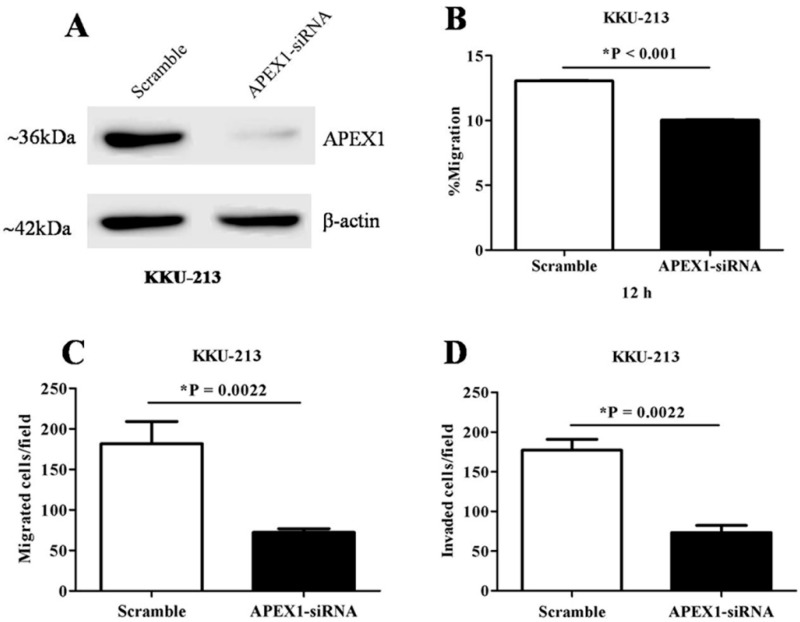
The effects of APEX1 gene-silencing on the CCA cell line KKU-213. (**A**) Western blot analysis showing suppressed APEX1 protein expression after gene silencing. β-actin was used as a control for loading protein. (**B**) Suppression of cell motility after APEX1 gene-silencing in wound healing model. (**C**) Suppression of cell migration after APEX1 gene-silencing in Transwell-migration assay. (**D**) Suppression of cell invasion after APEX1 gene-silencing in Matrigel-invasion assay.

To investigate the role of APEX1 in migration and invasion of CCA cells, we selected KKU-213 cell line of which APEX1 signal intensity level in cell lysate was much higher than the other two cell lines (Figure 6). Then, APEX1 gene of KKU-213 cell line was silenced using siRNA. The expression of APEX1 in KKU-213 was successfully suppressed (Figure 7A). Effects of APEX1 gene silencing on cell motilities were examined in vitro, using wound healing assay (Figure 7B), transwell migration assay (Figure 7C), and matrigel invasion assay (Figure 7D). Cell motility of APEX1-silenced KKU-213 was significantly lower than that of the scramble control in all three assays.

### 2.6. APEX1 Protein Interaction

**Figure 8:**
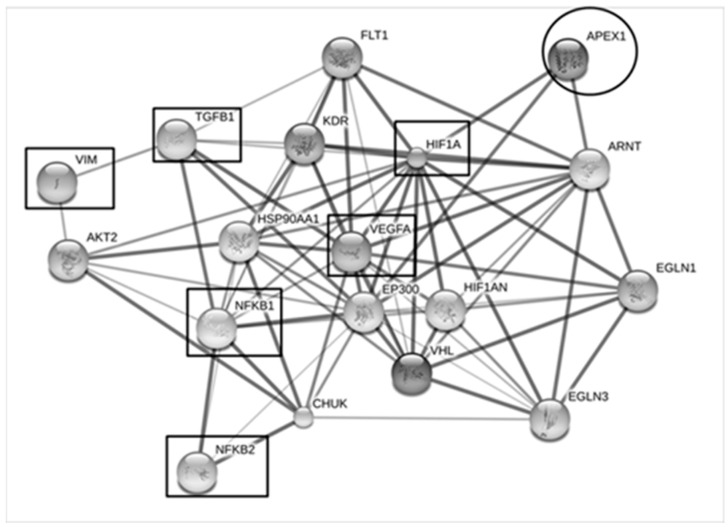
The interaction map of the APEX1 and metastatic processes-associated proteins. Protein-ligand interaction was predicted by STITCH Version 5.0. Protein-protein interactions are represented in solid lines. Stronger associations are represented by thicker lines. Weak associations are represented by thin lines. APEX1 was predicted to have strong interaction with vascular endothelial growth factor (VEGF), hypoxia-inducible factor-1alpha (HIF-1α), nuclear factor kappa B (NF-κB), transforming growth factor β (TGFβ), and vimentin (VIM).

To speculate potential roles of APEX1 in CCA metastasis mechanism, the possible interaction of APEX1 and metastatic proteins were predicted using STITCH version 5.0 (Figure 8). The results showed that vascular endothelial growth factor (VEGF), hypoxia-inducible factor-1 alpha (HIF-1α), nuclear factor kappa B (NF-κB), transforming growth factor beta (TGF-β), and vimentin (VIM) were identified as the protein molecules interacting with APEX1.

## 3. Discussion 

In this study, we used four bioinformatics tools, SignalP, SecretomeP, TMHMM, Plasma Proteome database, to select candidate proteins of secretory protein nature for the diagnosis of CCA. Bioinformatic analysis for the secretomes of three CCA cell lines and immortal cholangiocyte cell line revealed that APEX1, KRT19, and CLTB were the candidates for validation. When predicted signal peptide cleavage sites of these three proteins were analyzed using EMBOSS database, APEX1 was predicted to have three positions of signal peptide cleavage sites. In contrast, KRT19 and CLTB were predicted to have 0 positions of signal peptide cleavage sites. Thus, EMBOSS is useful for reducing the number of candidate proteins of possible secretory nature.

In the present study, APEX1 was shown to be a reliable diagnostic marker for CCA because APEX1 had higher sensitivity and specificity than CA19-9, CEA, and ALP, especially for metastatic CCA. The estimation of overall survival by Kaplan–Meier was significantly higher in patients with APEX1 low expression than in those with APEX1 high expression (Figure 5). Serum APEX1 was reported to be a biomarker for predicting prognosis and therapeutic efficacy of non-small cell lung cancer (NSCLC) [18]. It was reported that serum APEX1 level was higher in lymph node metastasis positive group than in the metastasis negative group of gastric cancer [19]. Also, serum and urinary APEX1 levels in bladder cancer patients of the late-stage were significantly higher than those in the early stage, and APEX1 level in the sera of muscle-invasive bladder cancer patients was higher than that in non-muscle invasive bladder cancer patients [20,21]. Huajun et al. (2018) showed that serum APEX1 autoantibody was higher in colorectal cancer group than that of the healthy control group. Moreover, sensitivity and accuracy of combined APEX1 and carcinoembryonic antigen-related cell adhesion molecule 1 (CEACAM1) in the diagnosis of colorectal cancer were significantly higher than individual detection of APEX1 or CEACAM1 [22]. Recently Kim et al. (2019) reported that APEX1 could be a reliable biomarker for the diagnosis of clear cell renal cell carcinoma and hepatobiliary carcinomas [17].

Until now, no definite tumor marker has been reported for CCA. In this study, we used secretome analysis and found that APEX1 was one of the major proteins secreted from CCA cells. As shown in Table 3, serum APEX1 seemed to be a better biomarker for CCA compared with previously known markers, such as CEA, CA19-9, or ALP. As APEX1 serum level has been reported to be increased also in various types of cancers, as mentioned above, serum APEX1 level should be used in combination with other diagnostic tools, such as ultrasonography and CT/MRI imaging for the diagnosis of CCA [18,19,20,21,22].

Interactions of APEX1 and metastasis mechanism-related proteins have been reported. For example, APEX1 was found to regulate transforming growth factor β-dependent manner to promote epithelial-mesenchymal transition (EMT) in osteosarcoma [23]. Furthermore, knockdown or inhibitor of APEX1 suppresses migration and invasion and promotes EMT through interaction with sirtuin-1 (SirT1) in non–small cell lung cancer (NSCLC) [24]. Wang et al. (2007) reported that APEX1 regulated vascular endothelial growth factor (VEGF) and fibroblast growth factor 2 (FGF2) through hypoxia-inducible factor-1α (HIF-1α) in osteosarcoma [25]. The APEX1 stimulates numerous transcriptional factors that are involved in cancer promotion and progression, such as HIF-1α, nuclear factor kappa B (NFκB) [26]. In this study, knockdown of APEX1 in KKU-213 cell line resulted in suppression of migration and invasion. 

In the previous study of liver cancer, several pathways have been shown to mediate the metastatic process. TGF-β expression is decreased in early, while increased in later stages of tumorigenesis [27,28]. Inhibition of TGF-β has been reported to up-regulate epithelial-cadherin (E-cadherin) and decrease migration and invasion [29]. Moreover, invasion and migration are through TGF-beta/Smad4 signaling pathway by epithelial-mesenchymal transition (EMT) [30]. Phoomak et al. reported that expression of matrix-metalloproteinase 7 (MMP7) was demonstrated to be of the NF-kB downstream signaling pathways that are involved with CCA cell migration/invasion [31]. As predicted by the STICH analysis in this study, these molecules might interact with APEX1 in association with metastatic process. Therefore, we performed additional experiments to see the effects of APEX1 protein level on cell migration and invasion.

## 4. Materials and Methods

### 4.1. Secretome Preparation and LC-MS/MS Analysis 

For the selection/prediction of candidate proteins from the panel of secreted proteins using bioinformatics tools, we have used the CCA secretome database constructed by Janan et al. (2012) [9], of which data were composed of the secretomes of three CCA cell lines, KKU-213, KKU-214, and KKU-100, and control immortalized cholangiocyte cell line, MMNK1. All cell lines in this study were confirmed to be mycoplasma-free by specific PCR. 

After precipitation by cold acetone, 50 µg of each protein sample was separated on 12.5% SDS-PAGE and stained with Coomassie Brilliant Blue R-250. Protein bands on each lane were cut into 15 segments according to size. In-gel digestion was performed, and tryptic peptide samples were injected in triplicate into anHCTUltra PTM Discovery LC-MS system (Bruker Daltonics Ltd; Hamburg, Germany), which was coupled with a nanoLC system: UltiMate 3000 LC System (Thermo Fisher Scientific; Madison, WI, USA), as well as an electrospray at the flow rate of 300 nL/min to a nanocolumn (PepSwift monolithic column 100 mm internal diameter x 50 mm). A mobile phase of solvent A (0.1% formic acid) and solvent B (80% acetonitrile and 0.1% formic acid) were used to elute peptides using multistep gradient of 10–70% of solvent B at 0–13 min (the time-point of retention), 90% solvent B at 13–15 min, followed by a decrease to 10% solvent B at 15–20 min. Peptide fragment mass spectra were acquired in data-dependent AutoMS mode with a scan range of 300–1500 *m/z;* 3 averages and up to 5 precursor ions selected from the MS scan of 50–3000 *m/z*.

DeCyderMS differential analysis software (DeCyderMS, version 2.0, GE Healthcare, Piscataway, NJ, USA) was used for the quantitation of peptides based on MS precursor signal intensities of individual LC-MS spectra. The quantitation of peptides was performed using the PepDetect module. Peptides were matched across different signal intensity maps between the control and treated samples using the PepMatch module. The relative abundances of peptides were expressed as log2 intensities. The analyzed MS/MS data from DeCyderMS were submitted for a database search using the Mascot software (Matrix Science, London, UK). The data were searched against the NCBI database for protein identification. Database interrogation was; taxonomy (Homo sapiens); enzyme (trypsin); variable modifications (carbamidomethyl, oxidation of methionine residues); mass values (monoisotopic); protein mass (unrestricted); peptide mass tolerance (1.2 Da); fragment mass tolerance (±0.6 Da); peptide charge state (1+, 2+, and 3+), and max missed cleavages (3) [9]. We selected proteins that were overexpressed in three CCA secretomes compared with MMNK1 secretome using the cut-off criteria of <0.05 in t-test and the log2 fold increase of MS signal intensities >1 as the first step of selection.

### 4.2. Serum Samples

Serum samples from 80 CCA patients (median age ± quartile deviation 61 ± 6.5 years; range: 38–79) and 39 serum samples from benign biliary diseases (BBD) consisting of cholangitis, cholecystitis, reactive hyperplasia, chronic inflammation, and cholelithiasis (median age ± quartile deviation 60 ± 7; range: 41–78) were collected from the Cholangiocarcinoma Research Institute, Faculty of Medicine, and Faculty of Associated Medical Sciences, Khon Kaen University, Thailand. The sera of CCA patients were all pre-operative cases, and prior to receive chemotherapy. The 40 serum samples from healthy control (median age ± quartile deviation of 52 ± 4.5; range: 40–59) were collected from those who came for a check-up at the Office for Medical Technology and Physical Therapy Health Service, Faculty of Associated Medical Sciences, Khon Kaen University. Biographical data, including liver functions of the participants, were summarized (Table 1). Healthy control group sera had normal liver function test. All serum samples were kept at −80 °C until use. This project was approved by the Human Ethics Committee of Khon Kaen University, Thailand (HE581431).

### 4.3. Prediction of Secretory Proteins

Secretory proteins were predicted using combination of five tools; SignalP to predict signal peptide cleavage sites using D-score >0.450 for the presence of a signal peptide within a protein sequence [32], SecretomeP to predict proteins with a neural network score (NN score) ≥0.5 for the proteins secreted via a non-classical secretion pathway without a signal peptide [33], Transmembrane hidden Markov model (TMHMM) 2.0 to predict transmembrane helix [34], and the Plasma Proteome Database (PPD) to identify proteins in serum or plasma [35]. European Molecular Biology Open Software Suite (EMBOSS) was used to predict signal peptide cleavage sites between a signal sequence and the mature exported protein [36]. Thus, the inclusion criteria included having predicted signal peptide or being predicted as non-classically secreted protein, together with having no transmembrane helix and being previously detected in plasma and having the highest number of signal peptide cleavage sites.

### 4.4. Western Blot Analysis

Fifty micrograms of protein samples of cell lysate were dissolved in sample buffer (10% sodium dodecyl sulfate (SDS), 1M Tris-HCl, pH 6.8), and boiled for 5 min. Protein concentration was determined by Bradford assay. The samples were separated on 12.5% SDS-PAGE at 135 V for 2 h at 4 °C. The samples were loaded and run in parallel with standard molecular weight markers. After electrophoresis, proteins were transferred onto PVDF membrane (GE Healthcare, Buckinghamshire, UK) for 1 h at room temperature. The membrane was blocked with 5% skimmilk in Tris-buffered saline with 0.1% Tween-20 (TBST, pH 7.4) for 1 h at room temperature. The membrane was then incubated with 1:500 dilution of rabbit polyclonal antibody against human APEX1 (Cat#orb129513, Biorbyt, Cambridge, UK) overnight at 4 °C. The membrane was washed with 1% TBST, incubated with 1:10,000 dilution of horseradish peroxidase-conjugated goatanti-rabbit IgG secondary antibody for 1 h at room temperature, and washed with 1% TBST. Finally, peroxidase activity was detected as chemiluminescence using an ECL plus reagent (GE Healthcare, Buckinghamshire UK) and quantitatively analyzed using an Image Quant LASmini4000.

### 4.5. Dot Blot Assay

The membrane was soaked in 1X Tris-buffered saline with 0.1% Tween-20 (TBST) for 10 min before placing on the machine. Two microliters each of serum samples were spotted onto a nitrocellulose membrane with Bio-Dot Microfiltration Apparatus (Bio-Rad, Hercules, CA, USA). To prepare a standard curve, APEX1 (Mybiosource, San Diego, CA, USA) with a known concentration was diluted two-fold as 1, 0.5, 0.25, 0.125, 0.0625, 0.0313, and 0.0156 pg/μL, respectively. For each assay, Hela cell lysate was used as a positive control for APEX1 protein. The membrane was then blocked with 5% skimmilk in 1X TBST for 1 h at room temperature. The membrane was then incubated with 1:500 dilution of primary antibody (rabbit polyclonal antibody against human APEX1) (Cat#orb129513, Biorbyt, Cambridge, UK) overnight at 4 °C. The membrane was washed with 1% TBST and then incubated with 1:10,000 dilution of horseradish peroxidase-conjugated goat anti-rabbit IgG secondary antibody for 1 h at room temperature, followed by washing with 1% TBST. Finally, the chemiluminescence was detected using ECL plus reagent (GE Healthcare, Buckinghamshire, UK) and quantified using an Image Quant LASmini4000. The intensities of APEX1 protein in the sera were normalized using APEX1 intensity in the Hela cell lysate as relative expression. Then, the relative expression of APEX1 in each serum sample was calculated based on the standard curve prepared using the standard APEX1 protein [37,38].

### 4.6. Transient Silencing of APEX1 Gene Using siRNA

Since APEX1 expression was highest in the cell lysate of KKU-213 cells in comparison with the cell lysate of the other two CCA cell lines, we selected this cell line for APEX1 gene silencing experiments. In brief, KKU-213 cells were cultured in Ham’s F-12 medium supplemented with 10% fetal bovine serum, 100 U/mL of penicillin, and 100 μg/mL of streptomycin and incubated at 37 °C in 5% CO_2_ air atmosphere. The cells were subcultured every three or four days. For the APEX1 gene silencing using a siRNA technique, the cells (1.5 × 10^5^ cells/well) were seeded in a 6-well plate and cultured overnight before being transfected with 100 pM of siAPEX1 (Cat#orb260731, Biorbyt, Cambridge, UK), while scrambled siRNA (Invitrogen, Carlsbad, CA, USA) was used as a negative control. Transfection was carried out using Lipofectamine 2000 (Invitrogen, Carlsbad, CA, USA) according to the manufacturer’s instructions. After 6 h of transfection, the culture medium was changed to complete medium, and the plates were incubated at 37 °C until 48 h. To check the suppression of protein expression, the cells were harvested in lysis buffer and incubated at 4 °C for 10 min. The cell lysate was centrifuged at 20,000 × g (4 °C) for 30 min. The level of APEX1 protein was determined using western blot analysis with ß-actin as a loading control. APEX1-silenced and control scrambled cells were tested for migration and invasion assay in vitro.

### 4.7. Wound Healing Assay

APEX1-silenced and control scrambled KKU-213 cells were cultured for 48 h after knock-down of APEX1 gene by siRNA, and the in vitro ‘scratch’ wounds were created with a sterile 10 µL pipette tip [39]. The old medium was discarded, and the wells were washed twice with sterile PBS, and freshly prepared serum-free medium was added. The cells were cultured for further 12 h. The wound edges were imaged using an inverted microscope fitted with an objective lens of 5x. Images were captured at 0 and 12 h after wound formation [39]. The distance of the wound closure was measured in three independent wound sites per group. Relative cell motility was calculated as the percentage of the remaining cell-free area compared with the area of the initial wound. Values from three independent experiments were pooled and expressed as mean ± SE.

### 4.8. Transwell-Cell Migration Assay

Effect of APEX1-silencing on cell migration was determined using a Transwell 24-well plate with the chamber membrane filter of 8 μm pore size (Corning, Kennebunk, ME, USA). APEX1-silenced and control scrambled KKU-213 cells were loaded in the upper chamber at the cell density of 4 × 10^4^ cells/200 µL of serum-free medium. The lower chamber was filled with 600 µL of complete medium. After 24 h, migrated cells on the lower surface of the membrane were fixed with 25% methanol and stained with 0.4% SRB in 1% acetic acid for 15 min, destained in 600 μL of 1% acetic acid for 5 sec. The migrated cells were counted using an inverted microscope fitted with an objective lens of 10x. Six randomly selected fields for each membrane filter were counted [24].

### 4.9. Matrigel-Cell Invasion Assay

Invasion ability of APEX1-knocked down and control scramble KKU-213 cells was determined using a Transwell 24-well plate with Matrigel-coated membrane (Corning, Kennebunk, ME, USA). The upper chamber was loaded with 4 × 10^4^ cells/200 µL of serum-free medium. The lower chamber was filled with 600 µL of complete medium. After 24 h, invaded cells on the lower surface of the membrane were fixed with 25% methanol and further steps were followed the methods as previously mentioned in Transwell-Cell Migration Assay [24].

### 4.10. Protein Interaction Analysis 

The association of functions and networks for proteins were analyzed using “STITCH 5.0” [40]. Proteins names were input into a text box of protein name. Then, “Homo sapiens” was selected as the organism, and then we clicked to continue. The page showed the list of the names of proteins, and then we clicked to continue. The page showed a confidence view. Stronger associations are represented by the thicker lines. Protein-protein interactions are shown in solid lines, chemical-protein interactions in dashed lines, and interaction between chemicals in dotted lines. 

### 4.11. Statistical Analysis

The data were presented as median ± quartile deviation and the range (minimum to maximum). The different values among two and three sample groups were estimated using the Mann–Whitney and Kruskal-Wallis tests, respectively. The correlation between serum APEX1 levels and patients’ clinicopathological parameters were analyzed using Fisher’s exact test. *p* < 0.05 was considered to indicate a statistically significant difference. The cut-off values were calculated using the receiver operating characteristic (ROC) curve analysis, and we selected the cut-off value to give the highest sensitivity and specificity value. Kaplan–Meier analysis was used to estimate the overall survival time. The GraphPad Prism v.5 software (GraphPad Software Inc., La Jolla, CA, USA) was used for statistical analyses.

## 5. Conclusions

In conclusion, the present results clearly showed that serum APEX1 level could be used as a potential biomarker for differentiation of CCA from normal healthy control or BBD. Moreover, higher serum APEX1 level was associated with lymph node metastasis and shorter survival time of the patients, so serum APEX1 level might also be a potential biomarker for poor prognosis of CCA. Since overexpression of APEX1 was observed in several other cancers, serum APEX1 level of CCA patients should be compared with that of other cancer patients, especially hepatocellular carcinoma, pancreas cancer, or other metastatic liver cancers, etc. Besides, the function of APEX1 should be further analyzed in relation to the signal pathway for metastasis mechanisms.

## Figures and Tables

**Table 1:** Characteristics of the study subject groups.

Parameters	Healthy Control (HC)	Benign Biliary Disease (BBD)	Cholangiocarcinoma (CCA)	*p*-Value
(Normal range)	(n = 40)	(n = 39)	(n = 80)	
Age	52 ± 4.5	60 ± 7	61 ± 6.5	<0.001 ^£¥^
	(40–59)	(41–78)	(38–79)	
Cholesterol	195 ± 16	172 ± 55.5 ^a^	184 ± 29.5 ^b^	0.325
(127–262 mg/dL)	(146–243)	(50–364)	(88–2202)	
Total protein	NA	7.5 ± 0.8 ^c^	7.3 ± 0.7 ^d^	0.594
(6.5–8.8 g/dL)		(4.1–9.1)	(4.5–9.8)	
Albumin	NA	3.5 ± 0.7 ^a^	3.9 ± 0.4 ^d^	0.037 ^£¥^
(3.8–5.4 g/dL)		(1.6–4.9)	(2–5.3)	
Total bilirubin	NA	1.2 ± 6.3 ^a^	1 ± 1.8 ^d^	0.515
(0.25–1.5 mg/dL)		(0.3–28.5)	(0.2–32.4)	
Direct bilirubin	NA	0.3 ± 4.5 ^a^	0.5 ± 1.2 ^d^	0.734
(0–0.5 mg/dL)		(0.1–20.4)	(0–24.9)	
ALT	19 ± 3.8	48 ± 19 ^a^	42 ± 36 ^d^	<0.001 ^£¥^
(4–36 U/L)	(8–33)	(8–537)	(2–283)	
AST	25 ± 2.8	50 ±33.5 ^a^	46 ± 40 ^d^	<0.001 ^£¥^
(12–32 U/L)	(14–31)	(16–577)	(11–1447)	
ALP	69.5 ± 9.8	174 ± 179 ^a^	158 ± 91.5 ^d^	<0.001 ^£¥^
(42–121 U/L)	(45–106)	(32–991)	(24–1963)	
Serum APEX1 levels	0.015 ± 0.016	0.024 ± 0.028	0.328 ± 0.352	<0.001 ^£¥#^
(pg/μL)	(0.010–0.064)	(0.012–0.218)	(0.015–0.901)	

Value represents median ± quartile deviation and (min-max). a, b, c, and d represent the number of analyzed subjects = 37, 73, 36, and 75, respectively. NA, not analyzed; ALT, alanine aminotransferase; AST, aspartate aminotransferase; ALP, alkaline phosphatase. The different values among two and three independent sample groups were estimated using the Mann-Whitney and Kruskal-Wallis tests, respectively. £ = Significant difference between HC and BBD. ¥ = Significant difference between HC and CCA. # = Significant difference between BBD and CCA.

**Table 2:** The overview of ROC curve evaluation of APEX1 as a diagnostic marker for CCA.

Discrimination between	Cut off Value (pg/μL)	Sensitivity (%)	Specificity (%)	Accuracy (%)	Positive Predictive Value (%)	Negative Predictive Value (%)	AUC (95%CI)	*p*-Value
Healthy controls vs. BBD	0.018	66.7	62.5	63.8	62.8	64.9	0.748	0.0001
BBD vs. CCA	0.050	90.0	84.6	87.5	91.1	80.5	0.927	<0.0001
Healthy controls vs. CCA	0.040	91.3	95.0	92.5	97.3	84.4	0.966	<0.0001
Healthy controls and BBD vs. CCA	0.080	88.8	97.5	93.1	97.3	89.5	0.947	<0.0001

**Table 3:** The sensitivity and specificity values of currently used serum markers and APEX1 for detecting CCA patients.

Marker ^a^	Sensitivity (%)	Specificity (%)	BBD (n)	CCA (n)
APEX1	90.1	85.3	39	80
CA19-9	62.1	64.3	14	42
CEA	83.3	50.0	14	36
ALP	68.2	62.1	37	75
Combination of CA19-9 and APEX1	56.2	95.1	14	42
Combination of CEA and APEX1	75.1	93.0	14	36
Combination of ALP and APEX1	61.2	94.2	37	75
Combination of CA19-9, CEA, and ALP	35.3	93.1	14	36
Combination of CA19-9, CEA, ALP, and APEX1	31.4	99.2	14	36

^a^ Cut-off values: APEX1; 0.050 pg/μL, CA19-9; 37 U/mL, CEA; 2.5 ng/mL, ALP; 121 U/L.

**Table 4:** Correlation between serum APEX1 level and clinicopathological features of CCA patients.

Clinical Parameters.	No.	Serum APEX1 Level (pg/μL)		
		≤0.328	>0.328	*p*-Value ^a^
Gender				0.642
Male	51	27	24	
Female	29	13	16	
Age (years)				
Mean range				0.823
≤59	37	19	18	
>59	43	21	22	
Lymph node metastasis				*<0.001
No	40	36	4	
Yes	40	4	36	
CA19-9				0.118
Normal (≤37 U/mL)	18	6	12	
Abnormal (>37 U/mL)	24	15	9	
CEA				0.677
Normal (≤2.5 ng/mL)	6	3	3	
Abnormal (>2.5 ng/mL)	30	12	18	
ALT				0.491
Normal (4–36 U/L)	36	20	16	
Abnormal (>36 U/L)	39	18	21	
AST				0.054
Normal (12–32 U/L)	27	18	9	
Abnormal (>32 U/L)	48	20	28	
ALP				0.862
Normal (42–121 U/L)	23	12	11	
Abnormal (>121 U/L)	52	26	26	
Survival time (days)	72	569.8 ± 476.5 (n = 35)	337.2 ± 233 (n = 37)	* 0.009 ^b^

* Statistically significant correlation, Fisher exact test, ^a^ These variables were analyzed from two groups of APEX1 (low and high level groups), ^b^ The different values among two groups were estimated using unpaired t-test, Survival time represents mean ± SD, Median of serum APEX1 level in CCA group used to separate between low and high groups.

## Supplementary Materials

The following are available online at https://www.mdpi.com/2218-273X/9/9/413/s1, Figure S1: Shuffling and randomization of serum samples, Figure S2: Validation of the accuracy of dot blot quantification. Table S1: The relative expression level of 90 distinct proteins found in the secretome of three CCA cell line.
