# Supplementary material for: Serum Apurinic/Apyrimidinic Endodeoxyribonuclease 1 (APEX1) Level as a Potential Biomarker of Cholangiocarcinoma
Source: Biomolecules. 2019 Aug 26;9(9):413. doi: 10.3390/biom9090413 (PMC6770206; doi:10.3390/biom9090413)
Supplement: Supplementary file 1 [file biomolecules-09-00413-s001.pdf]

biomolecules MDPI

Supplementary Materials

Table S1. The relative expression level of 90 distinct proteins found in the secretome of three CCA cell lines

Protein name	Accession number	*CM_K100	*CM_213	*CM_214
36/8-8 fusion protein with epitope for anti-lectin antibody, partial	gi|930041	16.49528	15.02345	15.74417
A Chain A, Structure Of The Extremely Slow Gtpase Rab6a In The Gtp Bound Form At 1.8 Resolution	gi|110590290	14.53508	15.01717	14.54292
alpha-1A-adrenergic receptor	gi|177807	12.66517	14.44567	12.27674
ankyrin repeat domain-containing protein 30A	gi|115495445	13.77647	13.31265	11.63526
ankyrin repeat domain-containing protein SOWAHB	gi|71274172	15.44109	13.38481	13.98292
arginine--tRNA ligase, mitochondrial precursor	gi|197100773	14.82117	17.84168	15.0465
ATP synthase lipid-binding protein, mitochondrial	gi|332839211	15.71769	15.5358	16.34782
breast cancer anti-estrogen resistance 1	gi|44894163	16.60799	16.93113	16.71035
Calpain 2, large [catalytic] subunit precursor variant	gi|62089296	13.37815	13.02746	12.44804
cathepsin L-like protein 6	gi|205689966	14.47527	11.39769	13.36508
CD160 antigen precursor	gi|5901910	15.1294	15.42182	14.65666
Chain A, Crystal Structure Of Ape1 From Homo Sapiens In A New Crystal Form Complexed With A Ligand	gi|118138505	15.43663	15.34845	15.08808
Chain A, Crystal Structure Of Dual-specificity Tyrosine Phosphorylation Regulated Kinase 2 (dyrk2)	gi|261278907	13.36311	16.0878	14.95161
Chain A, Crystal Structure Of Human Grp78 (70kda Heat Shock Protein 5 BIP) Atpase Domain In Complex With Atp	gi|320089786	13.22928	15.10335	16.32319
Chain A, Crystal Structure Of The N-Terminal Domain Of The Gem Interacting Protein	gi|326634553	14.35959	15.11035	15.41963
Chain X, Crystal Structure Of Cd5_diii	gi|145580233	12.93882	16.60748	16.97222
chromosome 9 open reading frame 10, isoform CRA_b	gi|119583268	14.28282	16.69975	15.97957
clathrin light chain B isoform b	gi|6005995	14.93705	16.77456	15.2394
cytochrome P450, family 2, subfamily C, polypeptide 8, isoform CRA_b	gi|119570402	14.59279	13.96739	14.27665
Dolichyl-phosphate mannosyltransferase polypeptide 1, catalytic subunit	gi|14250108	17.53929	17.79125	17.35948
dynein light chain 4, axonemal variant	gi|62898259	14.17495	13.77493	13.38152

E3 ubiquitin-protein ligase UBR1	gi|28372497	12.68564	11.81851	12.67639
Em:AC008101.5	gi|47678257	14.1376	12.57015	13.22407
EWS-FLI-1 protein, partial	gi|440999	14.9617	14.68782	14.87153
FLJ16008 protein, isoform CRA_b	gi|119615716	21.54119	21.80018	19.43054
FLJ44048 protein, isoform CRA_a	gi|119631344	16.07028	13.69475	14.61417
geranylgeranyltransferase type I beta subunit	gi|56566048	13.26863	13.7597	12.31978

Table S1. The relative expression level of 90 distinct proteins found in the secretome of three CCA cell lines (cont.)

Protein name	Accession number	*CM_K100	*CM_213	*CM_214
glutamate decarboxylase-like 1, partial	gi|119584818	12.14496	13.98104	13.67402
hCG1647589	gi|119592886	13.90688	14.65617	15.42308
hCG1779312, isoform CRA_b	gi|119585744	11.58915	12.47362	11.10312
hCG1789065	gi|119585555	15.69816	16.47006	15.80147
hCG1791432, partial	gi|119604414	15.55173	17.40927	15.95174
hCG2002943, partial	gi|119575392	16.05036	16.89151	14.30464
hCG2002956, isoform CRA_a	gi|119571233	12.3055	13.35396	13.93986
hCG2012538	gi|119591237	14.5371	15.47484	16.6449
hCG2040199, partial	gi|119625937	16.3645	16.79536	15.57812
hCG2045008	gi|119572109	13.6475	14.03598	15.9137
hCG2045180	gi|119583868	12.35126	15.04611	16.12196
hCG39696	gi|119611710	14.98462	13.12009	14.48346
heat shock transcription factor 2 binding protein	gi|7768755	16.02707	16.02027	13.54229
HEATR1 protein, partial	gi|40850891	13.72422	11.82243	11.31077
high affinity cGMP-specific 3',5'-cyclic phosphodiesterase 9A isoform d	gi|48762722	12.23516	14.00236	14.31614
HIV-1 Tat interacting protein, 60kDa, isoform CRA_d	gi|119594837	13.15963	13.22937	13.13731
homeobox protein	gi|32393	10.47545	12.54459	14.47484

immunoglobulin heavy chain	gi|7161051	12.54176	13.45388	11.63028
keratin, type I cytoskeletal 19	gi|24234699	14.29918	13.71068	14.33445
KIAA0453 protein	gi|34328012	18.44062	17.17805	17.20647
KIAA0868 protein	gi|4240225	13.78654	16.34488	15.35233
KIAA1865 protein	gi|14017947	14.85106	12.51114	15.1262
LanClantibioticsynthetase component C-like 2	gi|47125269	13.98841	12.5044	12.59366
LOC401407	gi|51094807	13.43095	12.51729	12.44492
MDN1, midasin homolog (yeast), isoform CRA_a	gi|119568930	11.41546	12.2714	11.30998
MFHAS1 protein, partial	gi|40225967	11.1423	12.95014	10.53614
mitochondrial carrier homolog 2 variant	gi|62897755	13.43892	13.93524	13.16586

Table S1. The relative expression level of 90 distinct proteins found in the secretome of three CCA cell lines (cont.)

Protein name	Accession number	*CM_K100	*CM_213	*CM_214
mitogen-activated protein kinase kinasekinase 13 isoform 2	gi|334085274	10.35446	6.333631	13.6363
Miz-1 protein	gi|2230871	13.33889	15.12203	15.17534
myopalladin isoform a	gi|153792294	17.03742	17.24949	14.84067
nitric oxide synthase 2A (inducible, hepatocytes), isoform CRA_a	gi|119571433	13.13112	12.69239	13.61458
nuclear pore complex protein Nup214	gi|33946327	15.63103	17.7353	13.68803
PC2-glutamine-rich-associated protein	gi|14276857	16.33864	13.82987	13.20408
phosphoinositide-3-kinase, class 2, gamma polypeptide	gi|119616793	15.43725	16.72274	15.48644
phospholipase DDHD2 isoform 1	gi|256017245	10.61705	9.759878	13.61121
potassium voltage-gated channel subfamily H member 4	gi|6912446	11.53175	14.28612	11.56258
protein FAM149A	gi|54792131	15.56241	15.95045	15.5624
protein FAM35A	gi|148596930	13.42251	11.51472	13.79277
putative	gi|553734	16.18468	18.31718	18.51236
putative protein LG72	gi|23238179	14.91237	12.3948	12.8857

RB-binding protein	gi|6572291	13.71861	16.56904	15.57394
RNA helicase-related protein	gi|3435312	13.51677	14.49298	15.07614
schlafen family member 5	gi|145580600	13.04957	13.81962	13.08636
serine/threonine-protein kinase tousled-like 2 isoform A	gi|45643117	17.06889	16.79583	16.73005
SHC-transforming protein 4	gi|222446609	13.82511	15.2448	14.91872
signal peptide, CUB and EGF-like domain-containing protein 2 isoform X2	gi|578820663	15.73411	14.44385	14.8858
SIPA1L2 protein, partial	gi|52078387	11.36655	12.18505	12.63254
solute carrier family 22 member 11 variant, partial	gi|62897297	15.11705	14.69114	15.24186
sperm-associated antigen 17	gi|46240864	15.53398	15.5738	15.75004
STAG3-like	gi|31417946	15.10803	11.7943	14.01262
suppression of tumorigenicity 18 protein	gi|7662168	10.75027	14.35599	10.67153
TDG protein	gi|116283610	17.86831	17.73831	17.61465
testis calpastatin	gi|3068550	11.10629	9.550511	14.16164
testis protein	gi|13161081	11.89292	15.23361	17.01753

Table S1. The relative expression level of 90 distinct proteins found in the secretome of three CCA cell lines (cont.)

Protein name	Accession number	*CM_K100	*CM_213	*CM_214
TMEM22 protein	gi|18490935	11.43042	15.14186	12.26173
TNFAIP3-interacting protein 3 beta	gi|146411668	9.222518	12.72582	13.86916
triosephosphate isomerase 1 variant	gi|62896835	13.84319	14.38596	14.35044
truncated MHC class I antigen	gi|184191069	12.74422	13.41399	12.53911
tyrosine-protein phosphatase non-receptor type 20 isoform 1	gi|108802604	12.93253	13.39809	14.14774
ubiquitin carboxyl-terminal hydrolase BAP1	gi|4757836	12.97787	12.97876	13.63815
UNC84B protein	gi|63102275	11.58352	13.94015	17.20445
Zinc finger protein 37 homolog	gi|116496933	12.81211	14.00895	14.00925
zinc finger, CCHC domain containing 14, isoform CRA_c	gi|119615797	8.714879	11.78555	11.80414

*Values represent the log2 fold change of precursor signal intensities in particular cell line secretome compared to immortalized human cholangiocyte secretome detected by LC-MS/MS

biomolecules

MDPI

11

12

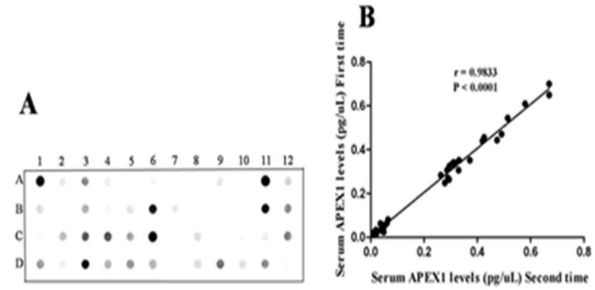

Figure S1 Shuffling and randomization of serum samples. (A) The result of dot blot. (B) The correlation of dot blot between first set and the second set of shuffled spotting.

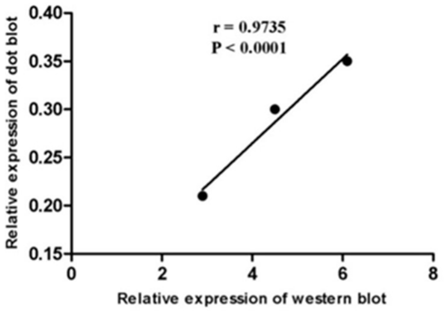

Figure S2 Validation of the accuracy of dot blot quantification.
The correlation between western blot and dot blot assay of relative expression of serum APEX1.
